# SPECC1L deficiency results in increased adherens junction stability and reduced cranial neural crest cell delamination

**DOI:** 10.1038/srep17735

**Published:** 2016-01-20

**Authors:** Nathan R. Wilson, Adam J. Olm-Shipman, Diana S. Acevedo, Kanagaraj Palaniyandi, Everett G. Hall, Edina Kosa, Kelly M. Stumpff, Guerin J. Smith, Lenore Pitstick, Eric C. Liao, Bryan C. Bjork, Andras Czirok, Irfan Saadi

**Affiliations:** 1 Department of Anatomy and Cell Biology, University of Kansas Medical Center, Kansas City, KS, USA; 2 Department of Biochemistry, Midwestern University, Downers Grove, IL, USA; 3 Center for Regenerative Medicine, Division of Plastic and Reconstructive Surgery, Massachusetts General Hospital, Harvard Medical School, Boston, MA, USA

## Abstract

Cranial neural crest cells (CNCCs) delaminate from embryonic neural folds and migrate to pharyngeal arches, which give rise to most mid-facial structures. CNCC dysfunction plays a prominent role in the etiology of orofacial clefts, a frequent birth malformation. Heterozygous mutations in *SPECC1L* have been identified in patients with atypical and syndromic clefts. Here, we report that in *SPECC1L*-knockdown cultured cells, staining of canonical adherens junction (AJ) components, β-catenin and E-cadherin, was increased, and electron micrographs revealed an apico-basal diffusion of AJs. To understand the role of SPECC1L in craniofacial morphogenesis, we generated a mouse model of *Specc1l* deficiency. Homozygous mutants were embryonic lethal and showed impaired neural tube closure and CNCC delamination. Staining of AJ proteins was increased in the mutant neural folds. This AJ defect is consistent with impaired CNCC delamination, which requires AJ dissolution. Further, PI3K-AKT signaling was reduced and apoptosis was increased in *Specc1l* mutants. *In vitro*, moderate inhibition of PI3K-AKT signaling in wildtype cells was sufficient to cause AJ alterations. Importantly, AJ changes induced by *SPECC1L*-knockdown were rescued by activating the PI3K-AKT pathway. Together, these data indicate SPECC1L as a novel modulator of PI3K-AKT signaling and AJ biology, required for neural tube closure and CNCC delamination.

Cranial neural crest cells (CNCCs) are specified in the dorsal neuroectoderm and delaminate from the neuroepithelium of the developing neural folds through a process that involves epithelial-mesenchymal transition (EMT)^1^,^2^,^3^. Premigratory epithelial CNCCs break down their cell-cell junctions and become migratory mesenchymal CNCCs, which populate the first and second pharyngeal arches, and give rise to the majority of craniofacial cartilage. Thus, genes that modulate CNCC function are frequently perturbed in the etiology of craniofacial congenital malformations such as orofacial clefts^3^,^4^,^5^,^6^,^7^, which are among the most frequent birth malformations affecting 1/800 births in the U.S. alone^8^.

CNCC delamination occurs simultaneously with anterior neural tube closure between mouse embryonic days 8.5 and 9.5. Many mouse mutants of genes associated with orofacial clefts also manifest some form of neural tube defects, including *Irf6*^9^,^10^*, Ghrl3*^10^*, Cfl1*^11^ and *Pdgfrα*^12^. However, the neural tube closure and CNCC delamination processes can be considered independent since the *Splotch* (*Pax3*) mouse mutant shows neural tube closure defects without any effect on CNCC delamination or migration^13^,^14^. Additional mouse models with defects in CNCC delamination and neural tube closure will help delineate the shared molecular underpinnings of these two processes.

Delamination of CNCCs from the neural epithelium requires dissolution of adherens junctions (AJs), composed of a protein complex, containing E-cadherin, β-catenin, α-E-catenin and α-actinin among others, tethered to actin filaments^2^. Studies where E-cadherin is overexpressed in the neural folds, show reduced or delayed delamination of CNCCs. Conversely, down-regulation of E-cadherin results in early delamination^15^,^16^. Many factors that mediate EMT during CNCC delamination are transcription factors (AP2α, Id2, FOXD3, SNAIL, TWIST, SOX10) and extracellular matrix (ECM) remodeling proteins such as matrix metalloproteases (MMPs), however, direct cytoskeletal regulators of CNCC AJs are not yet known. The PI3K-AKT pathway is known to antagonize E-cadherin levels, primarily from cancer studies^17^. Recent studies show that loss of murine PDGFα based PI3K-AKT signaling leads to craniofacial malformations including cleft palate and neural tube defects^12^. However, a link between the PI3K-AKT pathway and AJ stability in CNCC delamination is not clear.

Previously, we identified *SPECC1L* as the first gene mutated in two individuals with a severe cleft that extends from the oral cavity to the eye, termed Oblique Facial Cleft (ObFC) or Tessier IV clefts^18^. *SPECC1L* mutations have since been identified in two multi-generation families with autosomal dominant Opitz G/BBB syndrome (OMIM #145410), where affected individuals manifest hypertelorism and cleft lip/palate^19^, and in a family with Teebi hypertelorism syndrome (OMIM #145420)^20^. More than half of Opitz G/BBB syndrome cases are X-linked (OMIM #300000), caused by mutations in *MID1* gene^21^, which encodes a microtubule-associated cytoskeletal protein^22^. We proposed that SPECC1L, also a microtubule- and actin cytoskeleton-associated protein, may mediate transduction of signals required to remodel the actin cytoskeleton during cell adhesion and migration^18^. Using *in vitro* and *in vivo* studies, we now describe SPECC1L as a novel regulator of AJ stability through PI3K-AKT signaling. At the cellular level, SPECC1L deficiency resulted in reduced levels of pan-AKT protein and increased apico-basal AJ dispersion, which was rescued by chemical activation of the AKT pathway. *In vivo, Specc1l*-deficient embryos showed neural tube closure failure and reduced CNCC delamination. Thus SPECC1L functions in the highly regulated cell adhesion based signaling required for proper CNCC function during facial morphogenesis.

## Results

### SPECC1L is localized at cell boundaries in cultured cells upon confluency

**Figure 1:**
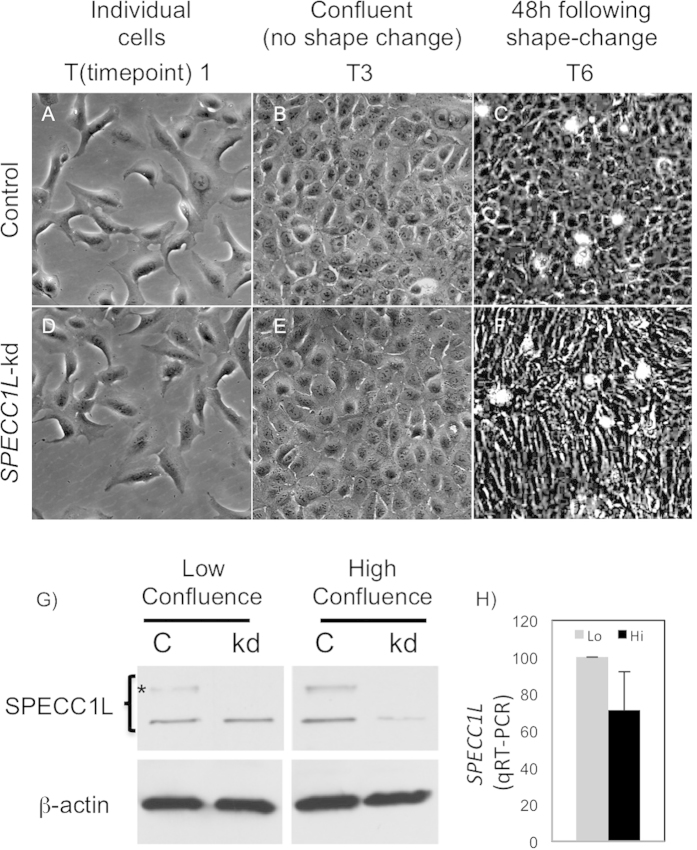
SPECC1L-knockdown cells elongate upon high confluency. (**A–F**) Compared to control U2OS cells (**A–C**), *SPECC1L*-knockdown cells (**D–F**) elongate upon high confluency (**F**). Three (T1, T3, T6) of the six time-points we chose for various cell densities are shown here. (**G**) Western blot analysis shows that SPECC1L protein is stabilized at high, compared to low confluency, in control cells. SPECC1L immunoblot shows an expected 120 kD band and a higher molecular weight band (*) that is likely modified post-translationally. The western blot analyses were performed under the same conditions for low and high confluence. The images shown for SPECC1L at low and high confluence are taken from a single blot. The same blot was stripped and re-probed with β-actin antibody. (**H**) Quantitative RT-PCR analysis shows no significant change in *SPECC1L* transcript levels. Error bars represent SEM from four independent experiments.

**Figure 2:**
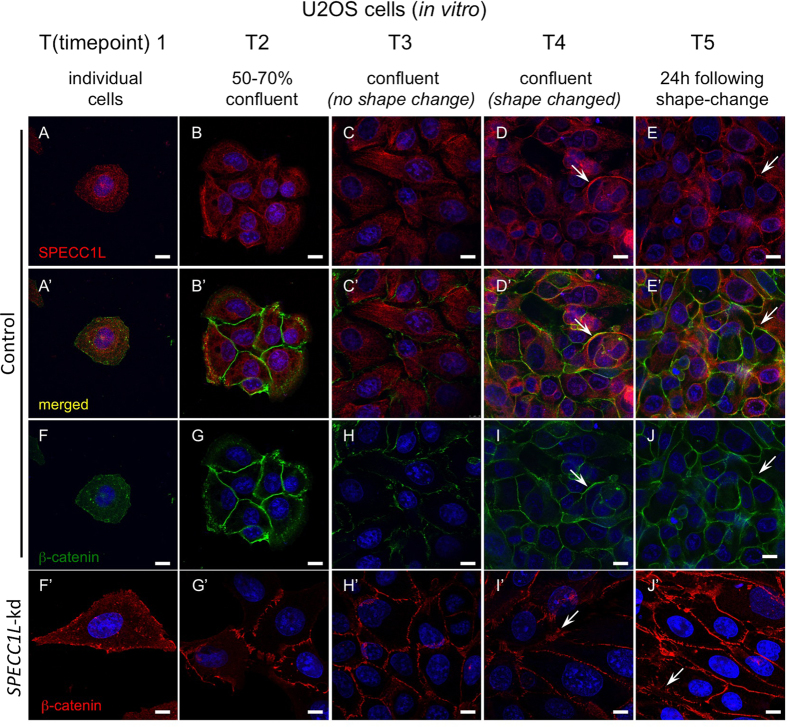
SPECC1L is stabilized at cell-cell boundaries similarly to β-catenin. (**A–E**) We picked six time-points (T1–T6) representing a range of cell densities to standardize analysis of cell shape and AJ change in *SPECC1L*-knockdown (kd) U2OS cells. First five of these time-points include individual cells (T1), 50–70% confluency with small cell clusters (T2), confluency without kd cell shape change (T3), shape change in kd cells (T4), and 24 hrs post shape change in kd cells (T5). SPECC1L protein is mostly dispersed within the cytoplasm at T1 (**A**), but is observed to accumulate at cell-cell boundaries at subsequent time-points (**B–E**), arrows). (**F–J**) β-catenin shows a similar accumulation at cell-cell boundaries in association with AJ complex. (**A’–E’)** SPECC1L and β-catenin show overlapping staining at cell boundaries at high cell densities (arrows). (**F’–J’)** In *SPECC1L*-kd cells, β-catenin staining appears normal at low cell densities (**F’**–**H’**), but is expanded upon cell shape change (I’,J’; arrows), indicating altered AJs. Bars = 10 μm.

To characterize the role of SPECC1L at the cellular level, we used the SPECC1L-deficient U2OS osteosarcoma stable cell line described previously^18^. These stable *SPECC1L*-knockdown (kd) U2OS cells have a moderate (60–70%) reduction in *SPECC1L* transcript and protein levels with defects in migration and actin cytoskeleton reorganization^18^. In contrast, a severe transient reduction in *SPECC1L* has been shown to cause mitotic defects^23^. Upon further characterization, we find that our stable *SPECC1L*-kd cells changed morphology upon very high confluency (Fig. 1). Individual control and kd cells at low confluency appeared similar (Fig. 1A,D). Twenty-four hours after confluency, control cells maintained their cuboidal shape (Fig. 1B,E), while *SPECC1L*-kd cells elongated (Fig. 1C,F). The extent of this cell shape change was captured by *in vivo* live-imaging of control and kd cells (Movie 1). To determine the role of SPECC1L in confluent cells, we first examined its expression. We found that SPECC1L protein level was increased upon confluency (Fig. 1G) without an increase in *SPECC1L* transcript levels (Fig. 1H). Furthermore, SPECC1L protein accumulated at cell-cell boundaries with increasing cell density (Fig. 2A–E), in a pattern overlapping with that of membrane-associated β-catenin (Fig. 2A’–E’). Given the association of SPECC1L with actin cytoskeleton^18^,^23^, we hypothesized that SPECC1L interacts with actin-based adherens junctions (AJs).

### SPECC1L deficient cells show abnormal staining and apico-basal dispersion of adherens junction proteins

**Figure 3:**
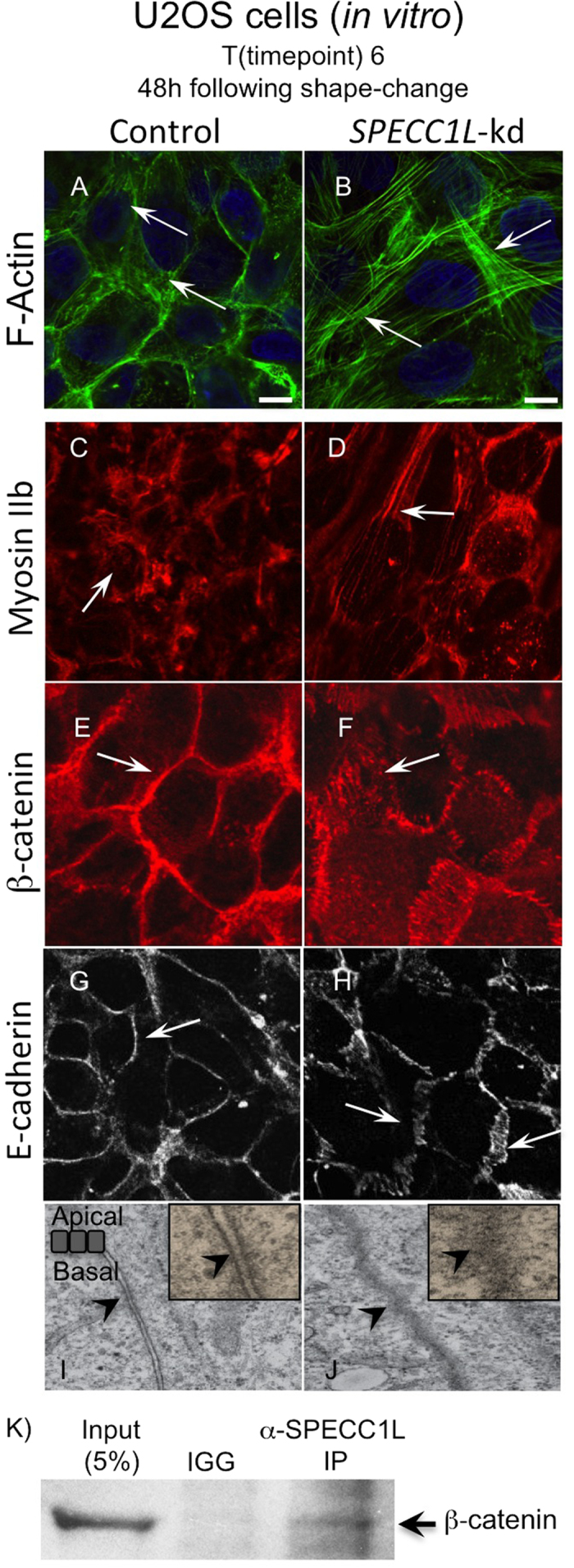
*SPECC1L*-kd U2OS cells show altered expression of adherens junction markers. (**A–H)** Increased F-actin staining in kd cells at 48 hours post-confluency (T6; **A,B**). Altered F-actin associated Myosin IIb staining (**C,D**). Smooth pattern of β-catenin and E-cadherin membrane staining in control cells (**E,G**) is expanded in *SPECC1L*-kd cells (**F,H**). Bars = 10 μm. (**I–J)** Electron micrographs looking at apico-basal cell-cell boundary. Control cells show distinct electron-dense regions, indicating adherens junctions (I, arrow). In contrast, the entire apico-basal boundary appears electron-dense in *SPECC1L*-kd cells (J, arrows), suggesting increased density and dispersion of adherens junctions. (**K**) β-catenin co-immunoprecipitation with *SPECC1L* in lysates from confluent U2OS cells. The image is taken from a single blot, and represents one of four independent experiments.

Next we sought to determine the effects of SPECC1L deficiency on AJs. We used several markers associated with AJs, including canonical components F-Actin, Myosin IIb, β-catenin, and E-cadherin^24^,^25^,^26^,^27^. As previously reported, actin stress fibers were increased in *SPECC1L*-kd cells (Fig. 3A,B)^18^. Myosin IIb, associated with actin filaments, showed a similar increase in *SPECC1L*-kd cells *in vitro* (Fig. 3C,D). AJ-associated β-catenin, which binds to cadherins at the cell membrane, showed a normal “honey-comb” pattern of expression in control cuboidal cells (Fig. 3E,G). Interestingly, in planar images using confocal microscopy, β-catenin (Fig. 3E,F) and E-cadherin (Fig. 3G,H) staining at the cell membrane in confluent SPECC1L-deficient cells showed a drastically expanded staining pattern. This expansion in AJ-associated β-catenin staining in kd cells was most evident upon confluency, but appeared to precede the cell shape change (Fig. 2F–J,F’–J’). To determine the physical nature of this expanded AJ staining, we examined the cell boundaries in the apico-basal plane of *SPECC1L*-kd U2OS cells (Fig. 3I,J) using transmission electron microscopy (TEM). Compared to control cells (Fig. 3I), which had distinct electron-dense regions indicating AJs (arrowheads), the kd cells (Fig. 3J) showed a large contiguous region of high electron density, suggesting extensive dispersion of AJs along the apico-basal plane. Also, in lateral sections, we observed widespread ruffling of the cell membrane in the kd cells (Fig. S1A,B), accounting for the expanded striated pattern of β-catenin and E-cadherin staining (Fig. 3F,H). In support of a role for SPECC1L in AJs, β-catenin co-immunoprecipitated with SPECC1L in lysates from confluent U2OS cells (Fig. 3K). Together with expanded immunostaining of AJ markers, the TEM analysis is consistent with our hypothesis that SPECC1L deficiency increased apico-basal density and dispersion of AJs.

### *Specc1l* deficiency leads to incomplete neural tube closure and reduced CNCC delamination

**Figure 4:**
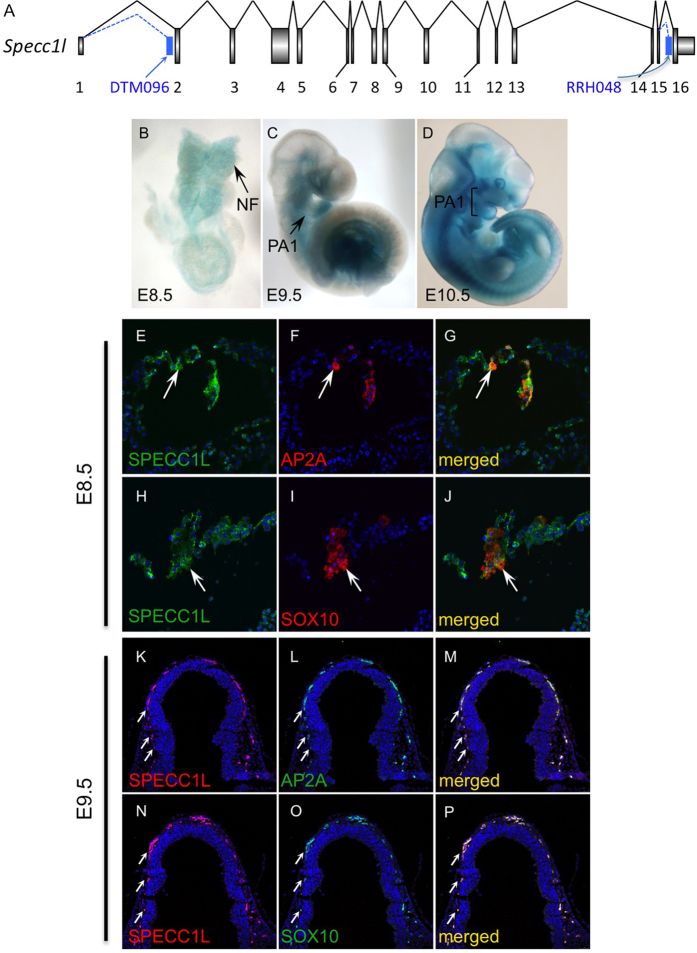
*Specc1l* expression and overlap with the neural crest cell lineage. (**A**) Schematic representation of murine *Specc1l* gene indicating insertion of genetrap vectors in ES cell clones DTM096 (intron 1) and RRH048 (intron 15). (**B–D)** Heterozygous *Specc1l*^*DTM096*^ embryos stained for *lacZ*, representing *Specc1l* expression, from E8.5 to E10.5. NE = neuroectoderm, NF = neural folds, PA1 = 1^st^ pharyngeal arch. (**E–P)** Co-immunostaining of SPECC1L with NCC markers AP2A and SOX10 in E8.5 neural folds (NF; **E–J**) and in E9.5 cranial sections (**K–P**). SPECC1L staining is broadly observed in the E8.5 neural folds (**E,H**; arrows), including in cells marked by AP2A (F,G; arrows) and SOX10 (**I,J**; arrows). At E9.5, SPECC1L strongly stains migrating CNCCs (**K,N**; arrows) marked by AP2A (**L,M**; arrows) and SOX10 (**O,P**; arrows).

To understand the role of SPECC1L in craniofacial morphogenesis, we created a mouse model of *Specc1l* deficiency using two independent gene-trap ES cell lines - DTM096 and RRH048 (BayGenomics, CA), which trap *Specc1l* transcripts in introns 1 and 15 respectively (Fig. 4A, Fig. S2). Genomic location of gene-trap vector insertion was identified by whole-genome sequencing and verified by PCR (Fig. S2). Both gene-trap constructs also afford in-frame *Specc1l-lacZ* reporter fusion upon trapping. Thus, *lacZ* expression, as determined by X-gal staining, was used as a proxy for *Specc1l* expression. Both alleles show a similar *lacZ* expression pattern with the DTM096 gene-trap in intron 1 showing stronger expression than RRH048 in intron 15 (not shown). *Specc1l* is expressed broadly, however, expression is particularly robust in the neural folds at E8.5 (Fig. 4B), the neural tube and facial prominences at E9.5 and E10.5 (Fig. 4C,D), and in the developing limbs and eyes at E10.5 (Fig. 4D). We previously reported that SPECC1L expression in the first pharyngeal arch at E10.5 is present in both the epithelium and the underlying mesenchyme^18^, consistent with CNCC lineage. To validate expression of SPECC1L in CNCCs, we co-stained for SPECC1L and NCC markers AP2-alpha (AP2A) and SOX10 in E8.5 neural folds (Fig. 4E–J) and in E9.5 cranial sections (Fig. 4K–P). At E8.5, SPECC1L stains the neural folds broadly (Fig. 4E,H), including cells stained with the NCC markers (Fig. 4G,J). At E9.5, SPECC1L (Fig. 4K,N) strongly stains migratory CNCCs co-stained with AP2A (Fig. 4L,M) or SOX10 (Fig. 4O,P).

Intercross matings between *Specc1l*^*DTM096/+*^ and *Specc1l*^*RRH048/+*^ heterozygous mice showed that the two gene-trap alleles failed to complement each other, and that compound heterozygous and embryos homozygous for either gene trap allele are embryonic lethal (Table S1). The Mendelian ratios indicate reduced survival of heterozygotes at birth (1.34 vs. 2.0 expected). We noted a low incidence of perinatal lethality in heterozygotes; some with craniofacial malformations (Fig. S3). However, the low penetrance of these perinatal craniofacial phenotypes makes it difficult to study the underlying pathophysiological mechanism. Therefore, we focused on the embryonic lethal phenotype of the *Specc1l* homozygous mutant.

**Figure 5:**
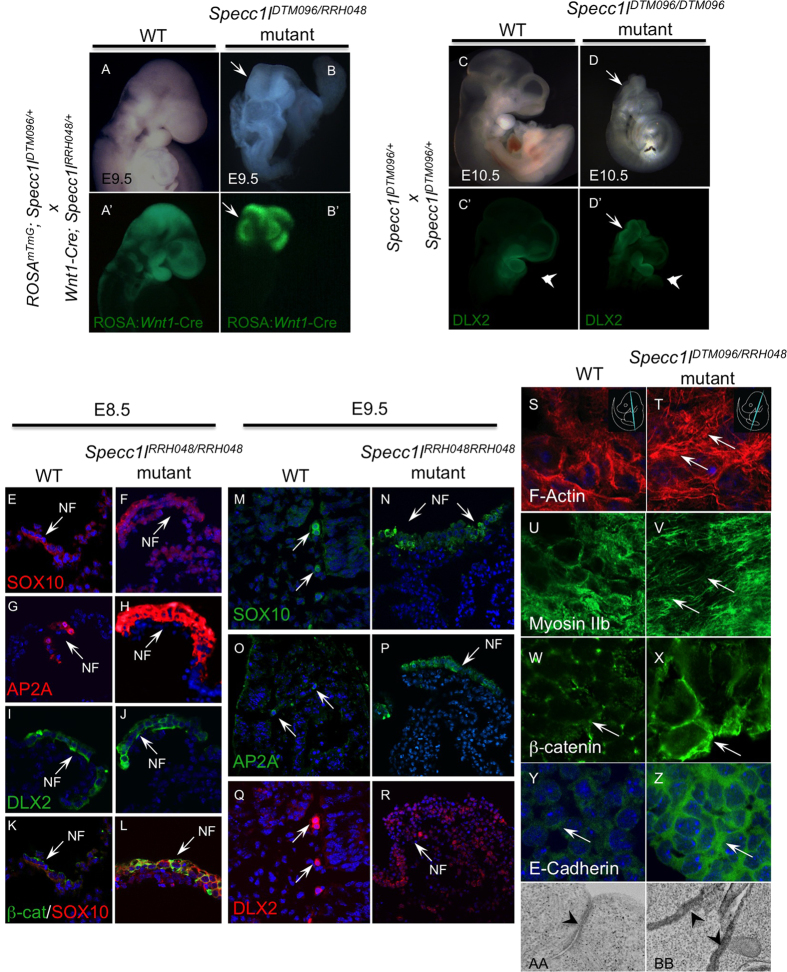
*Specc1l* deficiency leads to defects in neural tube closure, cranial neural crest cell delamination and AJs. (**A,B’)** E9.5 WT (**A**) embryo with migratory cranial neural crest cells (CNCCs) marked with *Wnt1*-Cre (**A**’). In contrast, *Specc1l* mutant embryo shows open neural folds (**B**), arrow) with CNCCs that have still not migrated (**B**’, arrow). (**C,D’)** Brightfield images (**C,D**) and CNCC marker DLX2 immuno-stain (**C’,D’**) of E10.5 WT (**C,C’**) and *Specc1l* mutant (**D,D’**) embryos. In E10.5 WT embryo, DLX2 positive CNCCs populate the branchial arches (**C’**, arrowhead), while in the mutant significant staining remains in open neural folds (**D’**, arrow) with some staining in the 1^st^ pharyngeal arch (**D’**, arrowhead), indicating poor CNCC delamination and migration. **E-R)** Sections from WT and *Specc1l* mutant embryos at E8.5 (**E–L**) and at E9.5 (**M–R**) were stained with NCC markers SOX10 (**E,F,M,N**), AP2A (**G,H,O,P**), and DLX2 (**I,J,Q,R**). At E8.5, NCC staining is observed in the neural folds (NF) of WT and mutant sections. Co-staining of SOX10 and β-catenin in E8.5 WT (**K**) and mutant (**L**) shows increased β-catenin staining at cell boundaries in the neural folds. At E9.5, migratory CNCC staining is observed in WT (**M,O,Q**), while in mutants undelaminated CNCCs stain the open neural folds (**N,P,R**). (**S–Z)** Analysis of AJ markers *in vivo* in WT and *Specc1l*^*DTM096/RRH048*^mutant E9.5 embryo coronal sections. The approximate plane of section is indicated in the top-right corner. Increased F-actin (**S,T**) and Myosin IIb (**U,V**) staining is observed in mutant tissue sections. Similarly to *in vitro* results in Fig. 3, expanded β-catenin (**W,X**) and E-cadherin (**Y,Z**) membrane staining is observed *in vivo* in mutant embryos. (**AA-BB)** Electron micrograph of WT embryo section looking at apico-basal cell boundary shows a distinct electron-dense region indicating adherens junction (**AA**, arrowhead). In contrast, the entire apico-basal boundary appears electron-dense in *Specc1l* mutant embryo section (**BB**, arrowheads), suggesting increased density and dispersion of adherens junctions.

The majority of *Specc1l*^*DTM096/RRH048*^compound heterozygous or homozygous mutant embryos failed to thrive beyond E9.5–10.5 (Fig. 5A–D), with a failure of the neural tube to close anteriorly (Fig. 5B,D), and sometimes posteriorly (not shown). This cranial neural tube closure defect was accompanied by a large proportion of CNCCs, marked by DLX2, to remain in the neural folds at E10.5, suggesting a failure to delaminate (Fig. 5A’–D’). To determine if there was also a reduction in overall specification of CNCCs, we marked the CNCC lineage with GFP in our gene-trap lines with *Wnt1-Cre* and *ROSA*^*mTmG*^. We flow-sorted the GFP+ NCCs and GFP- (RFP+) non-NCCs from whole embryos. At E9.5, the proportion of flow-sorted GFP-labeled CNCCs did not change significantly between WT and mutant embryos (not shown), indicating normal CNCC specification. Thus, we hypothesized that the residual staining with *Wnt1*-Cre and DLX2 in the open neural folds (Fig. 5B’) was due to a defect in CNCC delamination, possibly due to increased density or dispersion of AJs, as observed in *SPECC1L*-kd cells. We used NCC markers SOX10, AP2A, and DLX2 to confirm the presence of CNCCs in the neural folds (Fig. 5E–R). At E8.5, neural fold staining is observed with all three NCC markers in both WT (Fig. 5E,G,I) and *Specc1l* mutant (Fig. 5F,H,J) sections. At E9.5, while NCC markers stain migratory NCCs in WT sections (Fig. 5M,O,Q), residual NCC staining is present in the open neural folds of *Specc1l* mutant embryos (Fig. 5N,P,R). Since SOX10 and DLX2 mark migratory CNCCs, this result indicates that SPECC1L deficient CNCCs attain post-migratory specification but fail to emigrate from the neural folds.

### *Specc1l* mutant tissue recapitulates *in vitro* adherens junction changes

To test our hypothesis that reduced delamination is due to altered AJs, we investigated the AJ markers in the open neural folds of *Specc1l* mutant embryos (Fig. 5S–Z). We observed increased actin stress fibers (Fig. 5S,T) and concomitant increased localization of Myosin IIB staining to the actin fibers (Fig. 5U,V). Importantly, we saw increased staining of β-catenin (Fig. 5W,X) and E-cadherin (Fig. 5Y,Z) at cell-cell boundaries. We also examined β-catenin staining of NCCs in the neural folds of E8.5 embryos (Fig. 5K,L). The β-catenin staining appears more robust in *Specc1l* mutant neural folds (Fig. 5L vs. K), suggesting that changes in AJs have commenced. In electron micrographs of cranial sections from E9.5 embryos, we again observed an increase in dispersed electron-dense staining in *Specc1l* mutant embryos compared to WT (Figs 5AA,BB and S1E–H). Taken together, these results validate our *in vitro* findings in *SPECC1L*-kd U2OS cells, and indicate that abnormal staining of AJs precedes CNCC delamination in our mutant embryos.

### Reduced PI3K-AKT signaling and increased apoptosis in SPECC1L deficient cells

**Figure 6:**
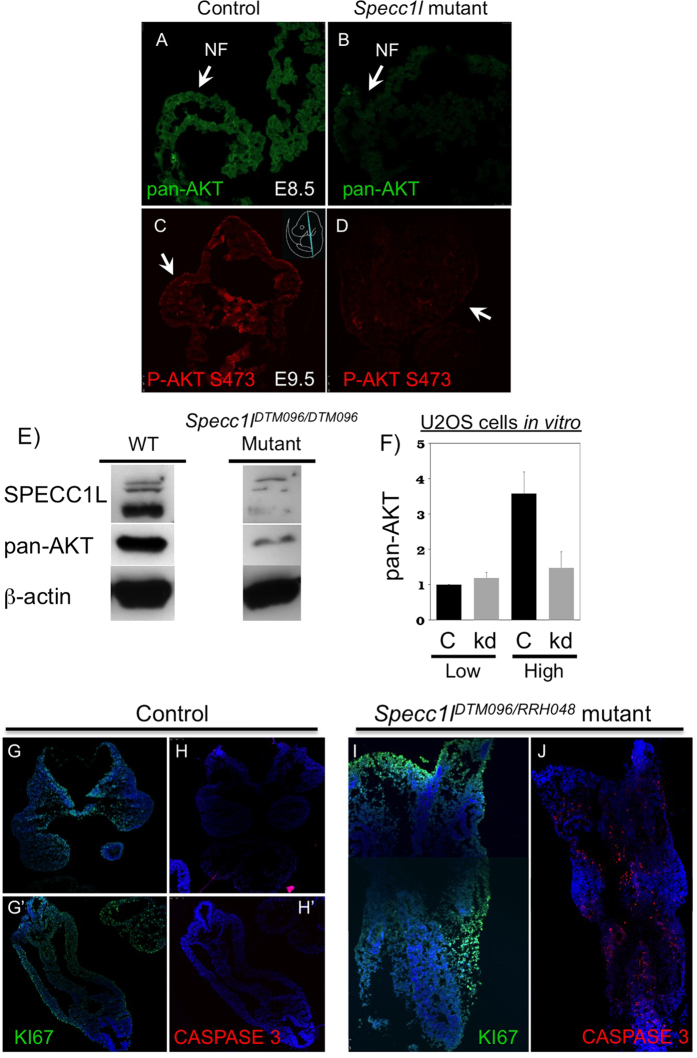
*Specc1l* mutant embryos show reduced PI3K-AKT signaling and increased cell death. (**A–E)** E8.5 (**A,B**) and E9.5 (**C,D**) cranial sections or E9.5 lysates (**E**) from *Specc1l* mutant embryos show decreased levels of active phospho-S473-AKT and pan-AKT protein, compared to WT control. The western blot analyses were performed under the same conditions for WT and mutant lysates. The images shown for SPECC1L are taken from a single blot. The same blot was stripped and re-probed with pan-AKT and then β-actin antibodies. Pan-AKT level in E8.5 neural folds (**A,B**) and phospho-S473-AKT level in E9.5 cranial section are markedly reduced. (**F**) A similar reduction is seen in pan-AKT levels in *SPECC1L*-kd U2OS cell lysates collected at high confluency. The error bars represent SEM from quantitation of three independent western blots. (**G–J**) Sections of WT embryos at E9.5 show cell proliferation (**G,G’**) with negligible apoptotic activity (H,H’) through KI67 and cleaved Caspase 3 staining, respectively. In *Specc1l* mutant embryos, there is comparable cell proliferation (I), however, there is a striking increase in cells undergoing apoptosis (**J**).

Given the known antagonistic relationship of AKT activity to E-cadherin stability^17^,^28^, we hypothesized involvement of PI3K-AKT signaling. In addition, we observed sub-epidermal blebbing in some of our mutant embryos that escape E9.5–10.5 lethality (<5%), and instead arrest at approximately E13.5 (Fig. S3). Sub-epidermal blebbing is a hallmark of reduced PDGFRα-based PI3K-AKT signaling^12^. Fantauzzo *et al*. (2014) reported that disruption of PDGFRα-based PI3K activation in *Pdgfra*^*PI3K/PI3K*^ mutant embryos results in sub-epidermal blebbing, neural tube defects, and cleft palate phenotypes. Indeed, pan-AKT and active phospho-Ser473-AKT levels were reduced *in vivo* in *Specc1l* mutant tissue (Fig. 6A–D), prior to embryonic arrest at E9.5. Reduced phospho-Ser473-AKT level was likely entirely due to reduced pan-AKT levels *in vivo* (Fig. 6E) and *in vitro* (Fig. 6F). The *in vitro* reduction was only present at high confluency of U2OS cells upon cell shape and AJ density changes (Fig. 6D). Thus, our data suggest SPECC1L is a novel positive regulator of PI3K-AKT signaling in craniofacial morphogenesis.

We next examined markers of proliferation and apoptosis. We did not observe any differences in proliferation in E9.5 embryos (Fig. 6E,G vs. I) as measured by KI67 staining, with a proliferative index of 82.5% for WT and 86.5% for *Specc1l* mutant (p < 0.56, Fisher exact test). Similarly, we did not observe any difference in apoptosis as measured by cleaved Caspase 3 staining in neural folds at E8.5, prior to embryonic arrest (not shown). In contrast, apoptosis was markedly increased throughout the E9.5 mutant embryo (Fig. 6F,H vs. J). This global increase in apoptosis is consistent with reduced PI3K-AKT signaling and early embryonic lethality^29^,^30^,^31^.

### Upregulation of PI3K-AKT pathway rescues *SPECC1L*-kd phenotype

**Figure 7:**
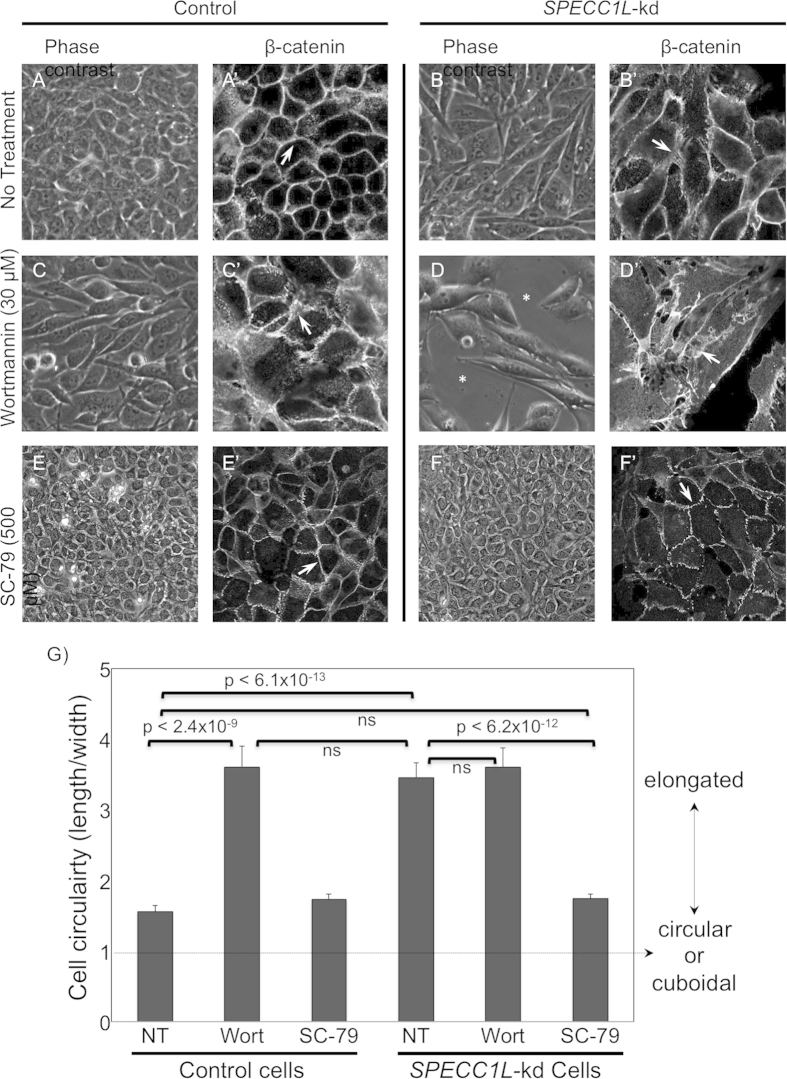
Upregulation of PI3K-AKT pathway can rescue *SPECC1L*-kd phenotype *in vitro*. (**A–F’)** Control (**A,C,E**) and *SPECC1L*-kd (**B,D,F**) cells were treated with PI3K-AKT pathway inhibitor Wortmannin (**C,D**) or activator SC-79 (**E,F**). Untreated control cells are cuboidal (**A**) with normal honey-comb pattern of β-cat staining (**A’**), while kd cells are elongated (**B**) with expanded β-cat staining (**B’**). Upon down-regulation of PI3K-AKT pathway, control cells are elongated (**C**) with β-cat expansion (**C’**), while kd cells begin to undergo apoptosis (**D**), similarly to our severe mutant embryos, and show extremely expanded β-cat staining (**D’**). Upon up regulation of PI3K-AKT pathway, control cells remain cuboidal (**E**) with normal β-cat staining (**E’**), while kd cells show much improved cell shape (**F**) and β-cat staining (**F’**), indicating rescue. (**G**) The extent of cell shape change in (**A–F**) were quantitated using a cell circularity ratio (CCR) of longest dimension (length) and the corresponding perpendicular dimension (width) using MetaMorph software. The no treatment (NT) *SPECC1L*-kd cells (CCR = 3.46) were significantly elongated than control cells (CCR = 1.56, p < 6.1 × 10^−13^). Wortmannin (Wort) inhibition of PI3K-AKT pathway in control cells was sufficient to cause a similar elongation of cell shape (CCR = 3.61, p < 2.4 × 10^−9^). Similarly, AKT activation with SC-79 in *SPECC1L*-kd cells was able to rescue the elongated cell shape back to control level (CCR = 1.74, p < 6.2 × 10^−12^). Wortmannin treatment of *SPECC1L*-kd cells resulted in increased apoptosis, but did not cause further increase in cell shape change (CCR = 3.60) beyond that observed in untreated kd (CCR = 3.46, ns) or Wortmannin-treated control cells (3.61). ns = not significant. Measurement of 50 cells +/− S.E.M. is shown. Statistical differences were calculated using a Student’s t-test.

**Figure 8:**
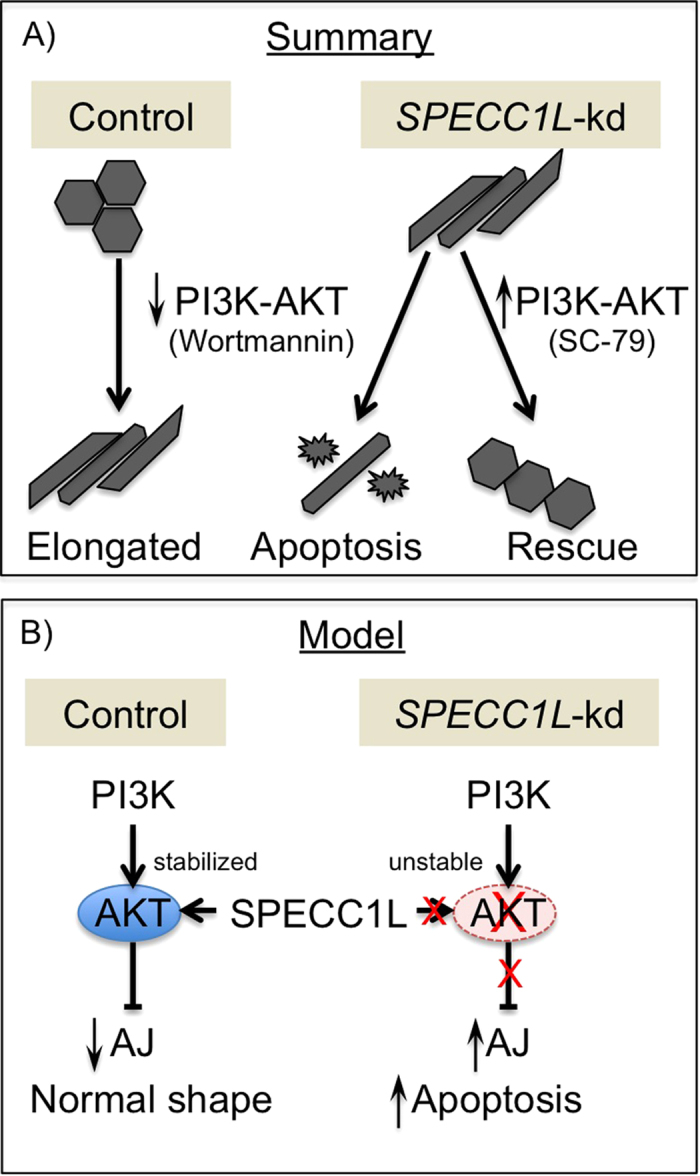
Model of SPECC1L modulation of PI3K-AKT pathway and AJs. (**A**) Schematic summary of PI3K-AKT pathway inhibition and activation leading to AJ change and rescue, respectively. (**B**) A model proposing that SPECC1L stabilizes AKT protein.

Next, to confirm a causal role for PI3K-AKT signaling in AJ change in our kd cells, we chemically altered the pathway in control and kd cells (Fig. 7A–F). We used the cell shape change phenotype observed in confluent *SPECC1L*-kd cells as a marker, which we quantified using the ratio of the longest dimension (length) and the corresponding perpendicular dimension (width). A relatively round or cuboidal cell would be expected to give a ratio of 1 (Fig. 7G). In addition to cell shape, we confirmed the effect on AJs through β-catenin staining (Fig. 7A’–F’). Inhibition of the PI3K-AKT pathway using Wortmannin was sufficient to alter cell shape (Fig. 7A,C) and AJs (Fig. 7A’) in control cells. The PI3K-AKT activator, SC-79, did not affect cell shape (Fig. 7A,E) or AJ expansion (Fig. 7A’) in control cells. In *SPECC1L*-kd cells, further down-regulation of PI3K-AKT pathway resulted in increased apoptosis (Fig. 7B,D) with drastically expanded staining for β-catenin (Fig. 7B’), consistent with our severe mutants *in vivo*. Importantly, upregulation of the PI3K-AKT pathway drastically ameliorated the cell shape (Fig. 7B,F) and AJ (Fig. 7B’’) phenotypes. As stated above, changes in cell shape were quantitated as a cell circularity ratio (CCR), and compared for significance (Fig. 7G). Indeed, in control cells (Fig. 7G, CCR = 1.56), Wortmannin treatment was sufficient to significantly change cell shape (Fig. 7G, CCR = 3.61, p < 2.4 × 10^−9^), which was similar in extent to that seen in *SPECC1L*-kd cells (Fig. 7G, CCR = 3.46). Wortmannin treatment of *SPECC1L*-kd cells (Fig. 7G, CCR = 3.60, not significant) did not affect cell elongation any further than untreated kd cells (Fig. 7G, CCR = 3.46, not significant) or Wortmannin-treated control cells (Fig. 7G, CCR = 3.61, not significant). Most important, SC-79 AKT activator rescued the elongated cell shape phenotype of *SPECC1L*-kd cells (Fig. 7G, CCR = 1.74, p < 6.2 × 10^−12^). These results substantiate that SPECC1L regulates PI3K-AKT signaling, and indicate that moderate reduction in SPECC1L affects cell adhesion, while severe reduction can lead to apoptosis (Fig. 8).

## Discussion

Dissolution of AJs is required for pre-migratory CNCCs to delaminate from the neural epithelium of the anterior neural folds^1^,^15^,^32^. The increased staining of AJ components and loss of apico-basal asymmetrical distribution of AJs in SPECC1L-deficient cells *in vitro* and *in vivo*, combined with physical proximity of SPECC1L with β-catenin indicates a role for SPECC1L in maintaining localized stability of AJs by properly organizing the actin cytoskeleton. The association of SPECC1L with the actin cytoskeleton and β-catenin combined with an increase in condensed actin filaments upon SPECC1L deficiency are consistent with the observed increase in AJ density. Another possibility is that increased actin fibers in SPECC1L deficient cells lead to altered tension between cells. As cell tension can impact AJ dynamics^33^, a change in tension may lead to more dispersed AJs^34^. Either alteration can consequently affect CNCC delamination.

*Wnt1* is expressed in the early neural folds that give rise to neural crest cells. Thus, *Wnt1*-cre lineage tracing marks both pre-migratory and migratory NCCs^35^. However, *Wnt1* also marks lineage of dorsal brain tissues that also arise from the early neural folds^35^,^36^, leaving a possibility that the *Wnt1*-marked staining in our E9.5 mutant open neural folds were not CNCCs. Our positive staining with NCC markers AP2A and SOX10 confirms that the open neural folds of the *Specc1l* mutant embryos indeed contain CNCCs. Furthermore, since AP2A and SOX10 are markers for early migrating NCCs, the positive staining indicates that these cells are post-migratory CNCCs that fail to delaminate by E9.5.

Our data indicate that the molecular regulation of AJs by SPECC1L is mediated by PI3K-AKT signaling. AKT signaling is reduced in SPECC1L-deficient cells and tissue. A direct role for PI3K-AKT signaling in craniofacial morphogenesis is supported by the findings of Fantauzzo *et al*. (2014) showing that lack of PDGFRα-based activation of PI3K-AKT signaling leads to cleft palate phenotype. We also showed that inhibition of the PI3K-AKT pathway is sufficient to alter AJs and cell shape in U2OS cells. Consistent with our findings, Cain *et al*.^37^ have shown that in endothelial cells down-regulation of PI3K α110 subunit led to a similarly expanded pericellular β-catenin staining, termed as increased “junctional index”. However, in their endothelial cells with already highly organized actin filaments, the down-regulation of PI3K-AKT pathway resulted in a relaxation of cell shape. In contrast, the *SPECC1L*-kd U2OS cells show elongated cell shape. This difference is likely cell-type specific. While down-regulation of PI3K-AKT signaling consistently affects the actin cytoskeleton, the effect on cell shape is dictated by altered tension resulting from changes in central actin fiber density and organization. In U2OS cells, we used the cell shape change only as a marker of the SPECC1L-deficient AJ change and rescue. Taken together, we propose that suppression of AKT pathway upon SPECC1L deficiency increases AJ stability and reduces delamination of the CNCCs.

Interestingly, pan-AKT levels are reduced in addition to phospho-473-AKT levels *in vitro* and *in vivo* upon SPECC1L deficiency, thus indicating regulation of PI3K-AKT signaling at the level of AKT protein stability or turnover. Both *SPECC1L* and *MID1* genes, implicated in Opitz/GBBB Syndrome, encode proteins that stabilize microtubules^18^,^22^. The mechanism through which SPECC1L and MID1 mediate microtubule stabilization is not entirely clear. In the case of SPECC1L, this stabilization involves enhanced acetylation of a subset of microtubules^18^. Perhaps SPECC1L utilizes a similar mechanism to stabilize other proteins such as AKT. Acetylation of lysine residues in AKT protein has been shown to result in reduced membrane localization and phosphorylation^38^. In addition, K63 chain ubiquitination of the same lysine residues on AKT is required for its membrane localization and activation^39^,^40^. Of the few SPECC1L protein interactors identified in different high throughput yeast two-hybrid screens, four-CCDC8^41^, ECM29^42^, APC and UBE2I^43^ - participate in protein turnover or stability through ubiquitination or sumoylation. It is possible that SPECC1L participates in the post-translational modification of AKT lysine residues, affecting AKT stability. However, a definitive role for SPECC1L in AKT protein localization and stability is yet to be elucidated.

A severe deficiency in SPECC1L expression *in vivo* results in increased AJ marker staining and defective CNCC delamination, as well as in increased apoptosis and early embryonic lethality. Previous reports have shown that mouse mutants with increased levels of apoptosis have associated neural tube^44^,^45^,^46^,^47^ and craniofacial defects^48^. It is proposed that excessive cell death in the neural folds or pharyngeal arches may result in insufficient number of cells required for proper morphogenetic movement^48^,^49^,^50^. In contrast, our SPECC1L-deficient cell lines with moderate reduction in *SPECC1L* expression show only AJ changes without evidence of increased cell death. However, chemical inhibition of PI3K-AKT pathway in these kd cells does indeed lead to increased apoptosis. Thus, a moderate reduction in SPECC1L expression or function allows cell viability. This is consistent with the observation that rare *Specc1l* mutant embryos that escape E9.5 arrest - likely due to reduced gene-trapping efficiency - are able to close their neural tubes and arrest later in development, frequently with craniofacial defects (Fig. S3). Also consistent with this is the rare occurrence of *Specc1l* heterozygous embryos with craniofacial malformation - likely due to increased gene-trapping efficiency - and findings in zebrafish where morphants for one of two *SPECC1L* orthologs (*specc1lb*) lead to late embryonic phenotypes including a loss of mandible and bilateral clefts^51^. Thus, heterozygous *SPECC1L* loss-of-function mutations identified in human patients may lead to mild perturbation in SPECC1L function during craniofacial morphogenesis, sufficient to account for their orofacial clefts. SPECC1L-based modulation of cell-cell contacts may also play a role in palatogenesis and pharyngeal arch fusion. Further studies of SPECC1L function will help elucidate the role of transient cell-cell contacts in neuroepithelial cell movement during neural tube closure and in CNCCs during craniofacial morphogenesis.

## Materials and Methods

### Cell lines and antibodies

U2OS osteosarcoma control and *SPECC1L*-kd cells were previously described (Saadi *et al*. 2011). Anti-SPECC1L antibody was also previously characterized (Saadi *et al*. 2011). Antibodies against β-catenin (rabbit; 1:1000; Santa Cruz, Dallas TX) (mouse; 1:1000; Cell Signaling Technology, Danvers, MA), Myosin IIb (1:1000; Sigma-Aldrich, St. Louis, MO), E-cadherin (1:1000; Abcam, Cambridge, MA), AP2A (1:1000; Novus Biologicals, Littleton, CO), SOX10 (1:1000; Aviva Systems Biology, San Diego, CA), DLX2 (1:1000; Abcam, Cambridge, MA), phospho-Ser473-AKT (1:1000; Cell Signaling Technology, Danvers, MA), pan-AKT (1:1000; ThermoFisher Scientific, Waltham, MA), KI67 (1:1000; Cell Signaling Technology, Danvers MA), cleaved Caspase 3 (1:1000; Cell Signaling Technology, Danvers, MA), and β-actin (1:2500; Sigma-Aldrich, St. Louis, MO) were used as described. Actin filaments were stained using Acti-stain rhodamine phalloidin (Cytoskeleton, Denver, CO).

### Cell shape change in U2OS cells

U2OS control and SPECC1L-kd cells were cultured in standard DMEM high glucose media supplemented with 10% fetal bovine serum (Life technologies, Carlsbad, CA). For AJ change, 2 × 10^5^ cells were plated on 0.1% porcine gelatin treated glass (Sigma-Aldrich, St. Louis, MO), and observed for cell shape change. Cells were collected at various indicated timepoints: 4 hours post plating (t = 1), 24 hours post plating (t = 2), confluency without cell shape change (t = 3), cell shape change (t = 4), 24 hours following cell shape change (t = 5), and 48 hours following cell shape change (t = 6) (Figs. 1, 2, 3). To modulate the PI3K-AKT pathway, cells were cultured in described concentrations of PI3K-AKT inhibitor, Wortmannin (TOCRIS Biosciences, Minneapolis, MN), or activator, SC-79 (TOCRIS Biosciences, Minneapolis, MN). Media with chemicals was replaced daily.

### Live-imaging analysis

Time-lapse recordings were performed of live control and kd cells in regular culture conditions with a phase contrast image collected every 10 minutes over a period of seven days. Images were taken with a computer-controlled Leica DM IRB inverted microscope equipped with a powered stage and a 10 × N-PLAN objective, coupled to a QImaging Retiga-SRV camera. During imaging, cell cultures were kept at 37C in a humidified 5% CO2 atmosphere.

### Mouse strains

Two gene trap ES cells lines, DTM096 and RRH048, from Mutant Mouse Regional Resource Center (UC Davis, CA) were used to generate *Specc1l* deficient mouse strains, designated *Specc1l*^*gtDTM096*^ and *Specc1l*^*gtRRH046*^. Briefly, 129/REJ ES cells were injected into C57BL6 blastocysts. The resulting chimeric male mice were crossed to C57BL6 females to identify progeny with agouti coat color. Presence of gene trap vector insertion was used to identify heterozygotes. The mice are maintained on a mixed 129/REJ; C57BL6 background. The location of the genetrap vector insertion site was verified by RT-PCR, genomic sequencing, and genetic complementation (Suppl. Fig. 1). For lineage-tracing of CNCCs, *Specc1l*^*GT*^ double heterozygous mice with ROSA^mTmG^ (#007576) and *Wnt1*-Cre (#003829) mice (Jackson Laboratories, Bar Harbor, ME) were intercrossed to generate *Specc1l* mutant embryos also carrying both ROSA^mTmG^ and *Wnt1*-Cre alleles. All mouse experiments were carried out in accordance with protocols approved by the Institutional Animal Care and Use Committee at the University of Kansas Medical Center.

### X-gal staining

Embryos were fixed in (1% formaldehyde, 0.2% gluteraldehyde, 2 mM MgCl_2_, 0.02% NP-40, 5 mM EGTA) for 60 minutes at room temperature. Following fixation, color development in X-gal staining solution (5 mM potassium ferricyanide, 5 mM potassium ferrocyanide, 2 mM MgCl_2_, 0.01% sodium deoxycholate, 0.02% NP-40, 1 mg/ml X-gal) was performed at 37 °C for 1–6 hours. Embryos were post-fixed in 4% PFA and imaged.

### Western blotting and immunostaining

For western blotting, cells were lysed in passive lysis buffer (Promega, Fitchburg, WI) with HALT protease inhibitor cocktail (Sigma-Aldrich, St. Louis, MO) added. Lysates were electrophoresed on 12% polyacrylamide Mini-PROTEAN TGX precast gels (Bio-Rad, Hercules, CA) and transferred onto an Immobilon PVDF membrane (EMD Millipore, Billerica, MA). Membranes were blocked in 5% milk in PBS with 0.1% Tween. Antibodies were incubated either overnight at 4 °C or for one hour at room temperature. Femto SuperSignal West ECL reagent (Thermo Scientific, Waltham, MA) was used to develop the signal. For immunostaining, embryos were fixed in 4% PFA/PBS overnight and cryopreserved. Tissue cryosections were blocked in PBS containing 1% normal goat serum (Thermo Scientific, Waltham, MA) and 0.1% Triton X-100 (Sigma-Aldrich, St. Louis, MO), followed by incubation in primary antibody overnight at 4 °C, and fluorescent secondary antibody (1:1000) for one hour at 4 °C. Stained sections were mounted in ProLong gold mounting media (Thermo Scientific, Waltham MA), and planar images were obtained using a Leica TCS SPE confocal microscope. Each immunostaining was performed as three independent experiments on cyrosections from at least two mutant embryos. A representative experiment is shown.

### Coimmunoprecipitation

Cells were lysed in a modified RIPA buffer (20 mM Tris-HCl pH8.0, 1% NP-40, 130 mM NaCl, 10% glycerol, 2 mM EDTA, and HALT protease inhibitors (Sigma-Aldrich, St. Louis, MO)). Briefly, lysates were pre-cleared with protein G magnetic beads (Life technologies, Carlsbad, CA) followed by incubation with anti-SPECC1L antibody or IGG overnight at 4 °C. Protein G magnetic beads were used to pull-down SPECC1L and western blotting was performed using β-catenin antibodies described above. The co-IP experiment shown is representative of four independent experiments.

### TEM analysis

Fixed cultured cells or mouse embryonic tissues were provided to the University of Kansas Medical Center Electron Microscopy Core. Briefly, samples were embedded in EMbed 812 resin (Electron Microscopy Sciences, Ft. Washington, PA), polymerized at 60 °C overnight, and sectioned with a Leica UC7 ultramicrotome equipped with a diamond knife, at 80 nm. Sections were imaged using a JEOL JEM-1400 Transmission Electron Microscope equipped with a Lab6 gun, at 100 kV.

## Additional Information

**How to cite this article**: Wilson, N. R. *et al*. SPECC1L deficiency results in increased adherens junction stability and reduced cranial neural crest cell delamination. *Sci. Rep.* **6**, 17735; doi: 10.1038/srep17735 (2016).

## Supplementary Material

Supplementary Movie

Supplementary Information

## References

[b1] Le Douarin N. The neural crest . (Cambridge University Press, 1982).

[b2] Saint-Jeannet J.-P. Neural crest induction and differentiation . (Springer Science + Business Media; Landes Bioscience/Eurekah.com, 2006).

[b3] Trainor P. A. Neural crest cells: evolution, development and disease . (Elsevier/AP, 2014).

[b4] Cordero D. R. . Cranial neural crest cells on the move: their roles in craniofacial development. American journal of medical genetics. Part A 155A, 270–279, doi: 10.1002/ajmg.a.33702 (2011). 21271641 PMC3039913

[b5] Bolande R. P. Neurocristopathy: its growth and development in 20 years. Pediatr. Pathol. Lab. Med. 17, 1–25 (1997). 9050057

[b6] Mangold E., Ludwig K. U. & Nothen M. M. Breakthroughs in the genetics of orofacial clefting. Trends in molecular medicine 17, 725–733, doi: 10.1016/j.molmed.2011.07.007 (2011). 21885341

[b7] Minoux M. & Rijli F. M. Molecular mechanisms of cranial neural crest cell migration and patterning in craniofacial development. Development 137, 2605–2621, doi: 10.1242/dev.040048 (2010). 20663816

[b8] Dixon M. J., Marazita M. L., Beaty T. H. & Murray J. C. Cleft lip and palate: understanding genetic and environmental influences. Nature reviews. Genetics 12, 167–178, doi: 10.1038/nrg2933 (2011). PMC3086810 21331089

[b9] Ingraham C. R. . Abnormal skin, limb and craniofacial morphogenesis in mice deficient for interferon regulatory factor 6 (Irf6). Nat. Genet. 38, 1335–1340, doi: 10.1038/ng1903 (2006). 17041601 PMC2082114

[b10] Peyrard-Janvid M. . Dominant mutations in GRHL3 cause Van der Woude Syndrome and disrupt oral periderm development. Am J Hum Genet 94, 23–32, doi: 10.1016/j.ajhg.2013.11.009 (2014). 24360809 PMC3882735

[b11] Harris M. J. & Juriloff D. M. An update to the list of mouse mutants with neural tube closure defects and advances toward a complete genetic perspective of neural tube closure. *Birth defects research*. Part A, Clinical and molecular teratology 88, 653–669, doi: 10.1002/bdra.20676 (2010). 20740593

[b12] Fantauzzo K. A. & Soriano P. PI3K-mediated PDGFRalpha signaling regulates survival and proliferation in skeletal development through p53-dependent intracellular pathways. Genes Dev. 28, 1005–1017, doi: 10.1101/gad.238709.114 (2014). 24788519 PMC4018488

[b13] Copp A. J., Greene N. D. & Murdoch J. N. Dishevelled: linking convergent extension with neural tube closure. Trends Neurosci. 26, 453–455, doi: 10.1016/S0166-2236(03)00212-1 (2003). 12948650

[b14] Murdoch J. N. & Copp A. J. The relationship between sonic Hedgehog signaling, cilia, and neural tube defects. Birth defects research. Part A, Clinical and molecular teratology 88, 633–652, doi: 10.1002/bdra.20686 (2010). PMC3635124 20544799

[b15] Taneyhill L. A. To adhere or not to adhere: the role of Cadherins in neural crest development. Cell adhesion & migration 2, 223–230 (2008). 19262148 10.4161/cam.2.4.6835 PMC2637483

[b16] Theveneau E. & Mayor R. Cadherins in collective cell migration of mesenchymal cells. Curr. Opin. Cell Biol. 24, 677–684, doi: 10.1016/j.ceb.2012.08.002 (2012). 22944726 PMC4902125

[b17] Larue L. & Bellacosa A. Epithelial-mesenchymal transition in development and cancer: role of phosphatidylinositol 3′ kinase/AKT pathways. Oncogene 24, 7443–7454, doi: 10.1038/Sj.Onc.1209091 (2005). 16288291

[b18] Saadi I. . Deficiency of the cytoskeletal protein SPECC1L leads to oblique facial clefting. Am J Hum Genet 89, 44–55, doi: 10.1016/j.ajhg.2011.05.023 (2011). 21703590 PMC3135813

[b19] Kruszka P. . Mutations in SPECC1L, encoding sperm antigen with calponin homology and coiled-coil domains 1-like, are found in some cases of autosomal dominant Opitz G/BBB syndrome. J. Med. Genet. , doi: 10.1136/jmedgenet-2014-102677 (2014). PMC4393015 25412741

[b20] Bhoj E. J. . Expanding the SPECC1L mutation phenotypic spectrum to include Teebi hypertelorism syndrome. American journal of medical genetics. Part A , doi: 10.1002/ajmg.a.37217 (2015). 26111080

[b21] So J. . Mild phenotypes in a series of patients with Opitz GBBB syndrome with MID1 mutations. American journal of medical genetics. Part A 132A, 1–7, doi: 10.1002/ajmg.a.30407 (2005). 15558842

[b22] Short K. M. & Cox T. C. Subclassification of the RBCC/TRIM superfamily reveals a novel motif necessary for microtubule binding. J. Biol. Chem. 281, 8970–8980, doi: 10.1074/jbc.M512755200 (2006). 16434393

[b23] Mattison C. P., Stumpff J., Wordeman L. & Winey M. Mip1 associates with both the Mps1 kinase and actin, and is required for cell cortex stability and anaphase spindle positioning. Cell cycle 10, 783–793 (2011). 21325884 10.4161/cc.10.5.14955 PMC3100791

[b24] D’Souza-Schorey C. Disassembling adherens junctions: breaking up is hard to do. Trends Cell Biol. 15, 19–26, doi: 10.1016/j.tcb.2004.11.002 (2005). 15653074

[b25] Stepniak E., Radice G. L. & Vasioukhin V. Adhesive and signaling functions of cadherins and catenins in vertebrate development. Cold Spring Harbor perspectives in biology 1, a002949, doi: 10.1101/cshperspect.a002949 (2009). 20066120 PMC2773643

[b26] Nishimura T. & Takeichi M. Remodeling of the adherens junctions during morphogenesis. Curr. Top. Dev. Biol. 89, 33–54, doi: 10.1016/S0070-2153(09)89002-9 (2009). 19737641

[b27] Strobl-Mazzulla P. H. & Bronner M. E. Epithelial to mesenchymal transition: new and old insights from the classical neural crest model. Semin. Cancer Biol. 22, 411–416, doi: 10.1016/j.semcancer.2012.04.008 (2012). 22575214 PMC3435443

[b28] Thomas-Tikhonenko A. Cancer genome and tumor microenvironment . (Springer, 2010).

[b29] Zeitlin S., Liu J. P., Chapman D. L., Papaioannou V. E. & Efstratiadis A. Increased apoptosis and early embryonic lethality in mice nullizygous for the Huntington’s disease gene homologue. Nat. Genet. 11, 155–163, doi: 10.1038/ng1095-155 (1995). 7550343

[b30] Mueller A. G. . Embryonic lethality caused by apoptosis during gastrulation in mice lacking the gene of the ADP-ribosylation factor-related protein 1. Mol. Cell. Biol. 22, 1488–1494 (2002). 11839814 10.1128/mcb.22.5.1488-1494.2002 PMC134710

[b31] Gladdy R. A., Nutter L. M., Kunath T., Danska J. S. & Guidos C. J. p53-Independent apoptosis disrupts early organogenesis in embryos lacking both ataxia-telangiectasia mutated and Prkdc. Molecular cancer research: MCR 4, 311–318, doi: 10.1158/1541-7786.MCR-05-0258 (2006). 16687486

[b32] Theveneau E. & Mayor R. Can mesenchymal cells undergo collective cell migration? The case of the neural crest. Cell adhesion & migration 5, 490–498, doi: 10.4161/cam.5.6.18623 (2011). 22274714 PMC3277782

[b33] Harris A. R., Daeden A. & Charras G. T. Formation of adherens junctions leads to the emergence of a tissue-level tension in epithelial monolayers. J. Cell Sci. 127, 2507–2517, doi: 10.1242/jcs.142349 (2014). 24659804 PMC4043320

[b34] Engl W., Arasi B., Yap L. L., Thiery J. P. & Viasnoff V. Actin dynamics modulate mechanosensitive immobilization of E-cadherin at adherens junctions. Nature cell biology 16, 587–594, doi: 10.1038/ncb2973 (2014). 24859003

[b35] Chai Y. . Fate of the mammalian cranial neural crest during tooth and mandibular morphogenesis. Development 127, 1671–1679 (2000). 10725243 10.1242/dev.127.8.1671

[b36] Brault V. . Inactivation of the beta-catenin gene by Wnt1-Cre-mediated deletion results in dramatic brain malformation and failure of craniofacial development. Development 128, 1253–1264 (2001). 11262227 10.1242/dev.128.8.1253

[b37] Cain R. J., Vanhaesebroeck B. & Ridley A. J. The PI3K p110alpha isoform regulates endothelial adherens junctions via Pyk2 and Rac1. J. Cell Biol. 188, 863–876, doi: 10.1083/jcb.200907135 (2010). 20308428 PMC2845076

[b38] Sundaresan N. R. . The deacetylase SIRT1 promotes membrane localization and activation of Akt and PDK1 during tumorigenesis and cardiac hypertrophy. Science signaling 4, ra46, doi: 10.1126/scisignal.2001465 (2011). 21775285

[b39] Yang W. L. . The E3 ligase TRAF6 regulates Akt ubiquitination and activation. Science 325, 1134–1138, doi: 10.1126/science.1175065 (2009). 19713527 PMC3008763

[b40] Chan C. H. . Posttranslational regulation of Akt in human cancer. Cell Biosci 4, 59, doi: 10.1186/2045-3701-4-59 (2014). 25309720 PMC4192732

[b41] Hanson D., Stevens A., Murray P. G., Black G. C. & Clayton P. E. Identifying biological pathways that underlie primordial short stature using network analysis. J. Mol. Endocrinol. 52, 333–344, doi: 10.1530/JME-14-0029 (2014). 24711643 PMC4045235

[b42] Gorbea C. . A protein interaction network for Ecm29 links the 26 S proteasome to molecular motors and endosomal components. J. Biol. Chem. 285, 31616–31633, doi: 10.1074/jbc.M110.154120 (2010). 20682791 PMC2951235

[b43] Bandyopadhyay S. . A human MAP kinase interactome. Nature methods 7, 801–805 (2010). 20936779 10.1038/nmeth.1506 PMC2967489

[b44] Homanics G. E. . Exencephaly and hydrocephaly in mice with targeted modification of the apolipoprotein B (Apob) gene. Teratology 51, 1–10, doi: 10.1002/tera.1420510102 (1995). 7597652

[b45] Ikeda A., Ikeda S., Gridley T., Nishina P. M. & Naggert J. K. Neural tube defects and neuroepithelial cell death in Tulp3 knockout mice. Hum. Mol. Genet. 10, 1325–1334 (2001). 11406614 10.1093/hmg/10.12.1325

[b46] Ruland J. . Bcl10 is a positive regulator of antigen receptor-induced activation of NF-kappaB and neural tube closure. Cell 104, 33–42 (2001). 11163238 10.1016/s0092-8674(01)00189-1

[b47] Migliorini D. . Mdm4 (Mdmx) regulates p53-induced growth arrest and neuronal cell death during early embryonic mouse development. Mol. Cell. Biol. 22, 5527–5538 (2002). 12101245 10.1128/MCB.22.15.5527-5538.2002 PMC133932

[b48] Jeong J., Mao J., Tenzen T., Kottmann A. H. & McMahon A. P. Hedgehog signaling in the neural crest cells regulates the patterning and growth of facial primordia. Genes Dev. 18, 937–951, doi: 10.1101/gad.1190304 (2004). 15107405 PMC395852

[b49] Copp A. J. Neurulation in the cranial region–normal and abnormal. J. Anat. 207, 623–635, doi: 10.1111/j.1469-7580.2005.00476.x (2005). 16313396 PMC1571567

[b50] Harris M. J. & Juriloff D. M. Mouse mutants with neural tube closure defects and their role in understanding human neural tube defects. *Birth defects research*. Part A, Clinical and molecular teratology 79, 187–210, doi: 10.1002/bdra.20333 (2007). 17177317

[b51] Gfrerer L. . Functional Analysis of SPECC1L in Craniofacial Development and Oblique Facial Cleft Pathogenesis. Plast. Reconstr. Surg. 134, 748–759, doi: 10.1097/PRS.0000000000000517 (2014). 25357034 PMC4430087

