# Supplementary material for: SPECC1L deficiency results in increased adherens junction stability and reduced cranial neural crest cell delamination
Source: Sci Rep. 2016 Jan 20;6:17735. doi: 10.1038/srep17735 (PMC4726231; doi:10.1038/srep17735)
Supplement: Supplementary Information [file srep17735-s2.pdf]

Supplementary Information

SPECC1L deficiency results in increased adherens junction stability and reduced cranial neural crest cell delamination

Nathan R. Wilson[1], Adam J. Olm-Shipman[1], Diana S. Acevedo[1], Kanagaraj Palaniyandi[1], Everett G. Hall[1], Edina Kosa[1], Kelly M. Stumpff[1], Guerin J. Smith[1], Lenore Pitstick[2], Eric C. Liao[3], Bryan C. Bjork[2], Andras Czirok[1], Irfan Saadi[1,4].

[1]Department of Anatomy and Cell Biology, University of Kansas Medical Center, Kansas City, KS.

[2]Department of Biochemistry, Midwestern University, Downers Grove, IL.

[3]Center for Regenerative Medicine, Division of Plastic and Reconstructive Surgery, Massachusetts General Hospital, Harvard Medical School, Boston, MA.

[4]To whom correspondence should be made:
isaadi@kumc.edu

Movie 1: *SPECC1L*-kd U2OS cells elongate upon high confluency. Equal number of Control (left) and *SPECC1L*-kd cells (right) were plated onto 35mm dishes and imaged. While control cells stay cuboidal, *SPECC1L*-kd cells elongate upon high confluency. Elapsed time is shown in hours.

Supplemental Table 1: *Specc1l*^{DTM} and *Specc1l*^{RRH} heterozygotes show reduced Mendelian ratio and homozygous mutants do not survive till birth.

**Analysis at birth:**		WT	Het	Hom	Litters
Specc1l$^{DTM/+}$ **x** *Specc1l*$^{DTM/+}$		29	43	0	13
Specc1l$^{DTM/+}$ **x** *Specc1l*$^{RRH/+}$		34	43	0	12
Specc1l$^{RRH/+}$ **x** *Specc1l*$^{RRH/+}$		16	20	0	8
	**Total**	**79**	**106**	**0**	**33**
**Mendelian Ratio**	**Observed**	**1**	**1.34**	**0**	
	**Expected**	**1**	**2**	**1**	

Wilson_Supplemental Figure 1

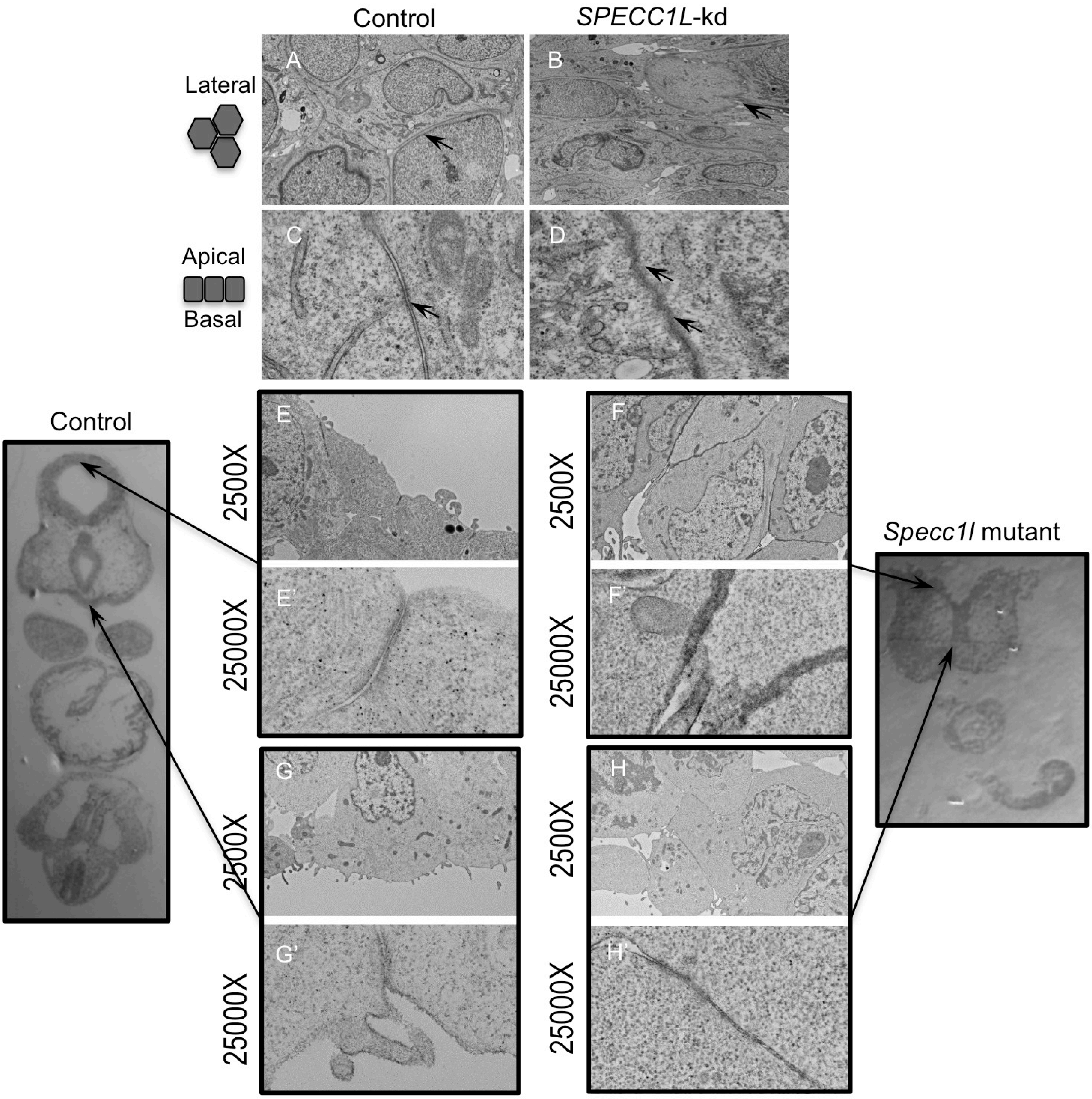

Figure S1: *SPECC1L*-kd cells and tissue show altered cell-cell boundaries in electron micrographs. Low magnification analysis of transverse sections show the normal honeycomb pattern for Control U2OS cells (A, arrow). However, *SPECC1L*-kd U2OS cells show jagged edges that appear intercalated (B, arrow). Higher magnification sections looking at apical-basal boundary shows distinct electron-dense regions indicating adherens junctions (C, arrow) In contrast, the entire apical-basal boundary appears electron-dense in *SPECC1L*-kd U2OS cells (D, arrows), suggesting increased density of adherens junctions. E-H) Importantly, the same increased density was noted *in vivo* in *Specc1l* mutant E9.5 embryo sections. Regions shown for control wildtype embryo (E,G) were taken from dorsal hindbrain (E) and maxillary pharyngeal arch (G) epithelia, while those for mutant embryos were taken from the open neural folds (F, H). Corresponding higher magnification images are shown in E'-H', indicating increased electron dense regions in the mutant cell-cell boundaries compared to those between control cells.

Wilson_Supplemental Figure 2

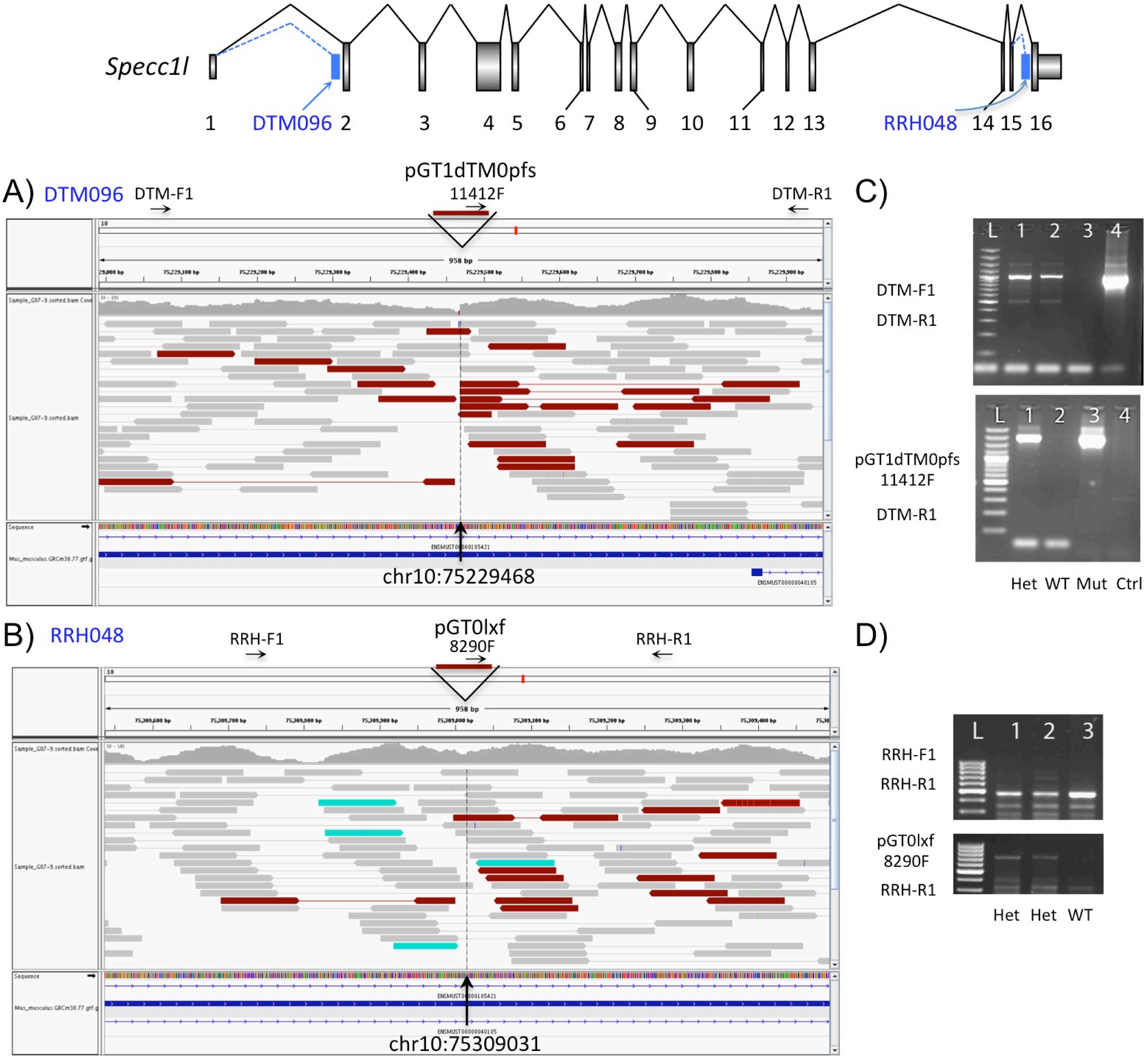

Primer Sequences		
	DTM-F1	GCTGCTTTGTGGCATTCTCA
	DTM-R1	CCACCACCTGCTTTCAGTCT
	pGT1dTM0pfs-11412F	CGTAAGGAGAAAATACCGCATCA
	RRH-F1	AGGGGCCATAAAATGCTGCT
	RRH-R1	GGGCTGTCCTGAGCAAGTTA
	pGT0lxf-8290F	ACCTCTGACACATGCAGCTC

Figure S2: Confirmation of genomic locations of *Specc1l* DTM096 and RRH048 genetrap constructs. Genomic DNA from a *Specc1l*$^{DTM096/RRH048}$ mutant embryo was analyzed by whole genome sequencing. As expected, disrupted mapping with clustering of un-mapped paired-ends was observed in *Specc1l* intron 1 and 15 corresponding to DTM096 (A) and RRH048 (B) reported locations. These insertions were confirmed by conventional PCR using a primer in the vector, thus establishing specific genotyping protocols for *Specc1l*^{DTM096} (C) and *Specc1l*^{RRH048} (D), respectively. Primer sequences and approximate locations are indicated for reference.

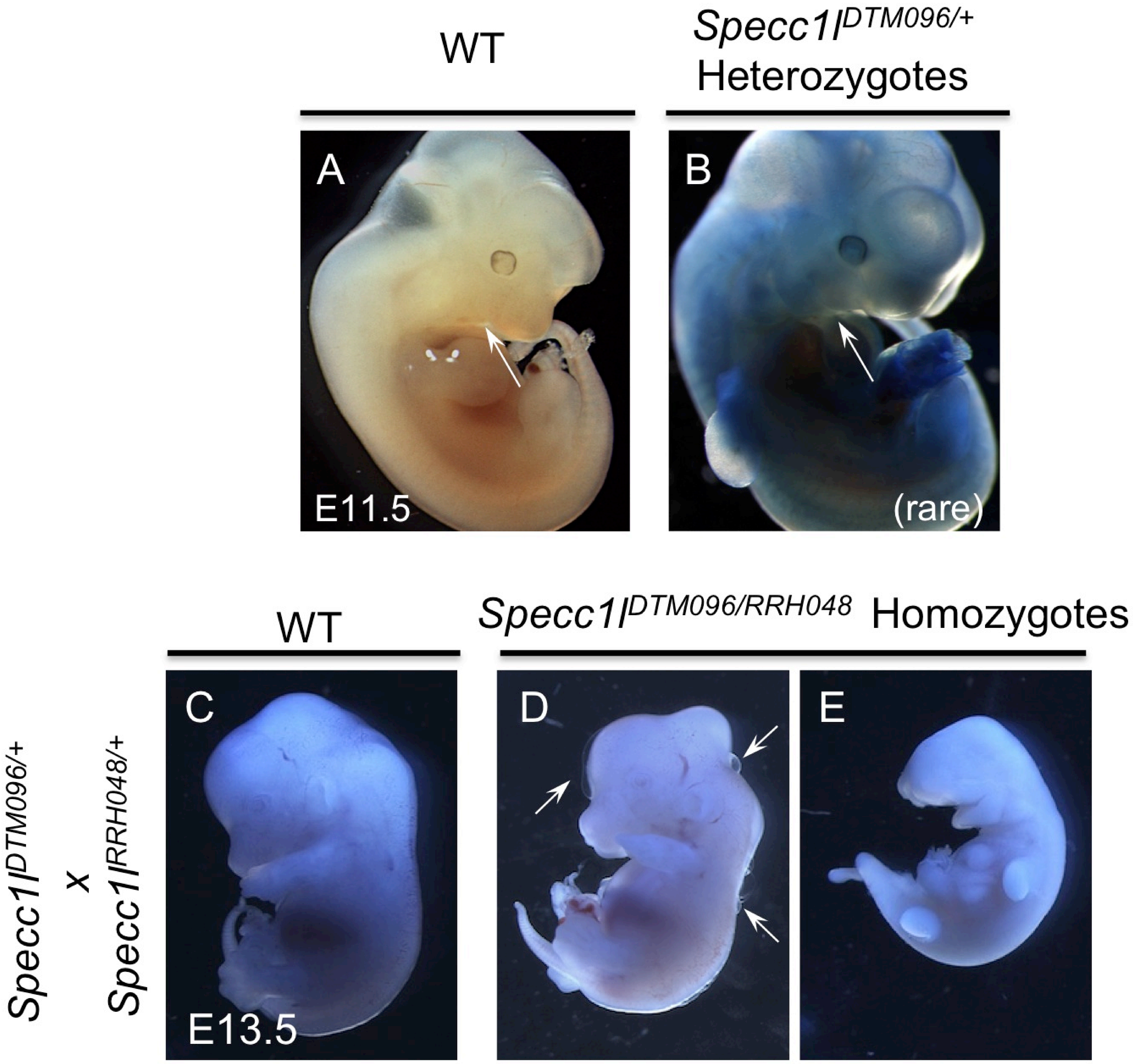

Figure S3: Rare late gestational phenotypes of heterozygous and homozygous *Specc1l* deficient embryos. A,B) In very rare cases, heterozygous *Specc1l* embryos show perinatal lethality. Shown are lacZ stained control 11.5 wildtype (A, clear) and a heterozygous littermate with hypoplastic facial processes (B, blue stained). C-E) In some cases, homozygous mutants escape early growth arrest and show a range of phenotypes including sub-epidermal blebbing (D, arrows) and severe craniofacial, limb and spine malformation (E), compared to WT littermate (C).
